# Congenital Aneurysm of the Right Atrium: Two Cases Report

**DOI:** 10.21470/1678-9741-2018-0047

**Published:** 2019

**Authors:** Halyna Melo, Fernando Moraes Neto, Cleusa Lapa, Carlos R. Moraes

**Affiliations:** 1 Instituto do Coração de Pernambuco, Real Hospital Português de Beneficência, Recife, PE, Brazil.

**Keywords:** Heart Atria/Pathology/Surgery, Aneurysm, Heart Defects, Congenital

## Abstract

Congenital aneurysm or enlargement of the right atrium is a rare condition. Two children operated on at the age of 14 months and 11 years old for congenital aneurysm of the right atrium are reported. Both presented cardiomegaly and symptoms of paroxysmal supraventricular tachycardia. Diagnosis was established by echocardiography. Surgical resection was successful. Both patients are free of symptoms and their chest X-ray and echocardiogram are normal. The first patient is now in her 17^th^ postoperative year. The patients' evolution suggests that the surgery is a curative procedure.

## INTRODUCTION

Congenital aneurysm or enlargement of the right atrium is a rare condition. It was firstly described and surgically corrected by Bailey^[1]^ in 1955. In an extensive survey of the international literature from 1955 to 1988, Binder et al.^[2]^ found 60 reported cases of right atrial aneurysm from which only 17 (28%) had been submitted to surgical treatment. Subsequently, sporadic cases have been described, including one of our experience^[3-6]^. In this article, we not only present another case, but probably more relevant, we describe the late results (17 years) of the surgery in the previously reported child.

## CASES REPORT

**Fig. 1:**
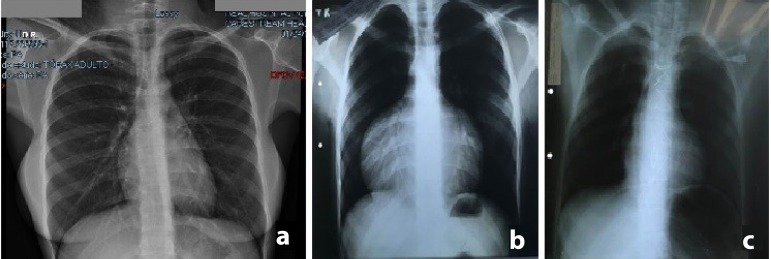
Chest X- rays of cases 1 and 2. Case 1: (a) Normal post-operative chest X-ray 17 years after surgery. Case 2: (b) Pre-operative chest X-ray showing marked cardiomegaly. (c) Normal post-operative chest X-ray.

Case 1 - A 14-months-old female patient was submitted to surgical excision of a right atrial aneurysm on March 30, 1999^[3]^. The diagnosis of congenital heart disease had been suspected by ultrasonography during fetal life. She was admitted to the emergency room of our institution presenting a paroxysmal supraventricular tachycardia which subsided with digoxin. Subsequently, she presented several episodes of arrhythmia. Physical examination was normal. Chest X-rays showed marked cardiomegaly. The electrocardiogram was normal. Echocardiogram demonstrated a massively dilated right atrium without any intracardiac abnormalities. Cineangiography confirmed the presence of a large aneurysm on the right atrium. Surgery was performed through a median sternotomy and normothermic cardiopulmonary bypass. The entire right atrium body was aneurismatic, but the atrial appendage was normal. The aneurysm was resected as much as necessary to simulate a normal-sized right atrium. The resected tissue measured 11 x 6 cm. The remaining right atrium was closed with a continuous 6-0 Prolene suture. The postoperative course was uneventful. She is now a 19-years-old health woman who had a normal pregnancy a year ago bearing a normal child. No episode of arrhythmia has occurred. Chest X-rays (Figure 1A), electrocardiogram, and echocardiogram are normal.

**Fig. 2:**
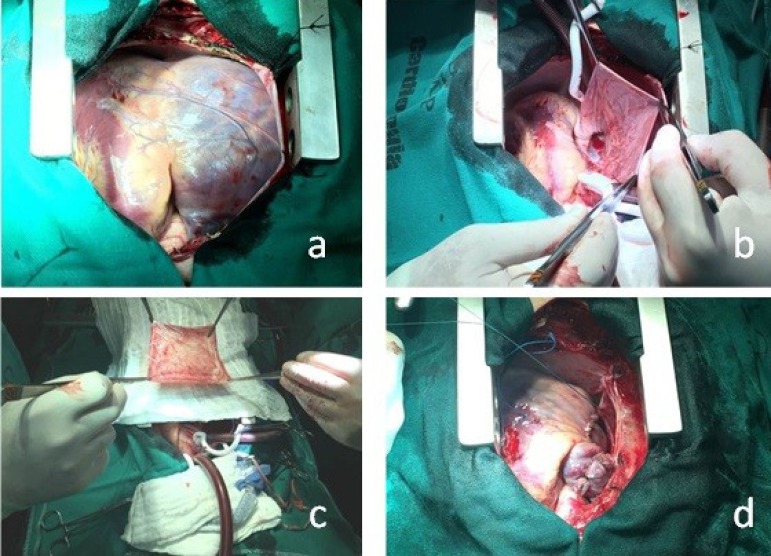
Intraoperative photographs of case 2. (a) View after the median sternotomy. (b) After establishment of extracorporeal circulation, the aneurysm sac is empty. (c) The resected right atrial tissue. (d) Heart after correction.

Case 2 - A 14-years-old male patient who had been diagnosed intra-uterus with congenital aneurysm of the right atrium was referred to our institution for surgical treatment. He had symptoms of frequent palpitations. Physical examination was normal, except for a systolic murmur grade 3/6 heard at the tricuspid area. The electrocardiogram was normal. The chest X-ray showed enlargement of the cardiac area (Figure 1B). Echocardiogram revealed aneurysmal dilatation of the right atrium and moderate tricuspid regurgitation. On November 24^th^, 2016, the patient underwent surgical correction (Figure 2). Under conventional cardiopulmonary bypass, the right atrial aneurysm was resected. The tricuspid valve was normal, but the annulus was dilated and a ring annuloplasty was performed. The postoperative course was uneventful. Chest X-ray (Figure 1C) and echocardiogram are normal.

## DISCUSSION

Congenital aneurysm of the right atrium also called idiopathic dilatation or congenital enlargement of the right atrium is rare. In our institution, over a period of 30 years (1987-2017), during which more than 23.000 cardiac operations have been performed, we have seen only 2 cases of such anomaly.

Right atrial aneurysms have been documented from neonatal to adult life^[2]^. In our cases, the suspicion of a cardiac anomaly was made during ultrasonography at gestation. Patients with right atrial aneurysms may be asymptomatic, but more frequently, as in our 2 children, they present supraventricular arrhythmias. This is probably the result of severe atrial dilatation since patients regained normal sinus rhythm after surgical removal of the aneurysm. Another possible complication is the formation of mural thrombus in the right atrium^[2]^. Massive dilatation of the right atrium may be associated with tricuspid annular dilatation and regurgitation requiring tricuspid annuloplasty. It did occur in our second patient.

In asymptomatic individuals, the diagnosis is suspected by the presence of cardiomegaly on chest radiography. Echocardiography may be the preferred method of diagnosis excluding other conditions such Ebstein's anomaly, pericardial effusion, pericardial cysts, and tumors. In a few cases, computed tomography, magnetic resonance imaging, and selective cineangiocardiography may be used to establish definitive diagnosis.

The analyses of the literature show that there is no unified opinion about the treatment of patients with aneurysm of the right atrium. Patients with arrhythmia have been treated surgically. Conservative approach is suggested to asymptomatic patients diagnosed with mild or moderate dilatation. We have advocated surgical treatment to all patients to prevent further dilatation of the right atrium and to avoid unexpected life-threatening complications. Moreover, the procedure has a low surgical risk^[3]^ and the late results of surgery observed in our first patient, 17 years after operation, suggest that resection of the right atrial aneurysm is a curative procedure.

**Table:** 

Authors' roles & responsibilities	
HM	Substantial contributions to the conception of the work; the acquisition and interpretation of data of the work; furthermore, agreement to be accountable for all aspects of the work in ensuring that questions related to the accuracy or integrity of any part of the work are appropriately investigated and resolved; final approval of the version to be published
FMN	Substantial contributions to the conception of the work; the analysis and interpretation of data for the work; final approval of the version to be published
CL	Agreement to be accountable for all aspects of the work in ensuring that questions related to the accuracy and integrity of any part of the work are appropriately investigated and resolved; final approval of the version to be published
CRM	Drafting the work and revising it critically for important intellectual content; final approval of the version to be published
